# Desmoglein1 Deficiency Is a Potential Cause of Cutaneous Eruptions Induced by Shuanghuanglian Injection

**DOI:** 10.3390/molecules23061477

**Published:** 2018-06-19

**Authors:** Yidan Zhang, Xiujun Zhang, Shanshan Fan, Lili Song, Zhen Yang, Pengwei Zhuang, Yanjun Zhang

**Affiliations:** 1 Chinese Materia Medica College, Tianjin University of Traditional Chinese Medicine, Tianjin 300193, China; zhangyidandandan@163.com (Y.Z.); fanshanshan420@163.com (S.F.); sll0204@163.com (L.S.); yzwygb@126.com (Z.Y.); 2 Tianjin Academy of Traditional Chinese Medicine, Tianjin 300120, China; tjzxj1982@163.com; 3 Tianjin State Key Laboratory of Modern Chinese Medicine, Tianjin University of Traditional Chinese Medicine, Tianjin 300193, China

**Keywords:** desmoglein1, cutaneous eruptions, keratinocyte, inflammation, apoptosis

## Abstract

Cutaneous eruption is a common drug-adverse reaction, characterised by keratinocytes inflammation and apoptosis. Shuanghuanglian injeciton (SHLI) is a typical Chinese medicine injection, which is used to treat influenza. It has been reported that SHLI has the potential to induce cutaneous adverse eruptions. However, the mechanisms remain unclear. Since desmoglein 1 (DSG1) shows a crucial role in maintaining skin barrier function and cell susceptibility, we assume that DSG1 plays a critical role in the cutaneous eruptions induced by SHLI. In our study, retinoic acid (RA) was selected to downregulate the DSG1 expression, and lipopolysaccharide (LPS) was first used to identify the susceptibility of the DSG1-deficiency Hacat cells. Then, SHLI was administrated to normal or DSG1-deficient Hacat cells and mice. The inflammatory factors and apoptosis rate were evaluated by RT-PCR and flow cytometry. The skin pathological morphology was observed by hematoxylin and eosin (HE) staining. Our results show that treated only with SHLI could not cause IL-4 and TNF-α mRNA increases in normal Hacat cells. However, in the DSG1-deficient Hacat cells or mice, SHLI induced an extreme increase of IL-4 and TNF-α mRNA levels, as well as in the apoptosis rate. The skin tissue showed a local inflammatory cell infiltration when treated with SHIL in the DSG1-deficient mice. Thus, we concluded that DSG1 deficiency was a potential causation of SHLI induced eruptions. These results indicated that keratinocytes with DSG1 deficiency were likely to induce the cutaneous eruptions when stimulated with other medicines.

## 1. Introduction

Cutaneous eruption, the most common drug-adverse reaction, refers to the skin reactions after drugs are delivered into the body through injection, oral administration, or inhalation [1,2]. With the development process of drug eruption, the release of inflammatory factors may increase. In addition, the apoptosis of keratinocytes is also the main histological features [3]. Keratinocytes, the most important cells during the development of cutaneous drug reactions, take part the skin barrier function and the production of the inflammatory mediators [4]. The apoptosis of keratinocytes may injure the barrier function of skin and increase cutaneous eruptions reaction [5]. An efficient skin barrier can prevent water loss and the entry of simulants and allergens [6,7]. When the barrier function of the skin is damaged, a lot of skin diseases will occur, such as atopic dermatitis and cutaneous eruptions.

Desmosome, including desmogleins (Dsgs) and desmocollins (Dscs), is a critical structure between cells and cells [8,9]. Desmosomal cadherin, a Ca^2+^-dependent adhesion protein, is a kind of signal protein, which can participate in the proliferation, differentiation, and morphogenesis of cells [10]. Desmogleins1 (DSG1), a member of the cadherin family, distributes through the spinous and granular layers. Its most important function is the ability to regulate cell adhesion and epithelial cell differentiation [11,12]. A large number reports show that the lack of DSG1 will damage the integrity of the skin epithelium, induce cell separation and lead to various dermatitis, multiple allergies, and metabolic wasting [13,14]. Besides, Tomoko Sumitomo and his colleagues’ findings suggested that the Desmogleins plays a pathogenic role in development of cutaneous infection [15]. Retinoic acid (RA) is a metabolite of vitamin A. Kim et al. reported that RA could decrease the DSG1 portein expression [16]. These results indicated that DSG1 could be a pharmacological intervention and an important target of drug induced cutaneous eruption. 

SHLI is an injectable lyophilized powder for intravenous injection approved by the Chinese FDA to treat upper respiratory tract infection. It is mainly composed of *Lonicera japonica Thunb*, *Coptis chinensis Franch*, and *Forsythia suspensa*, containing many components, such as chlorogenic acid, baicalin, forsythiaside, and caffeic acid [17]. It is reported that SHLI may cause cutaneous reactions when combined with other medicines or when used on the patients with an allergic constitution [18]. However, the mechanisms remain unclear. Yi et al. found that the kind of solvent (such as glucose and saline injection) and the drug preparation time may be the reasons for the hypersensitive responses caused by SHLI [19]. Other researchers considered the components of SHLI as the reason for allergic reactions [20]. Therefore, to improve the safety of clinical use of SHLI, it is important to reveal the real reason and potential mechanisms of the cutaneous reactions caused by SHLI. According to the important role of DSG1 in skin rashes, we considered that the DSG1 deficiency might be one of the potential reasons of the cutaneous reactions caused by SHLI. And we finally identified that DSG1 deficiency might play an important role in SHLI induced dermatitis medicamentosa. Because of the DSG1 deficiency could be hereditary or induced by pharmacological regulation, the present study could also provide a new research strategy on the drug safety evaluation.

## 2. Materials and Methods

### 2.1. RA induced Hacat cells DSG1 Deficiency

The cell line, Hacat (in vitro spontaneously transformed keratinocytes from histologically normal skin, cobioer biosciences, CBP60331), was cultured in a modified Eagle’s medium (MEM, HyClone, Logan, UT, USA), complete medium supplemented with 10% fetal bovine serum (FBS, Biological industries, Kibbutz, Israel), and 1% penicillin-streptomycin (HyClone, Logan, UT, USA) at 37 °C and 5% CO_2_. The medium was changed every day. When the cell density was about 90%, the Hacat cells were cultured in six-well plates for subsequent experiments. To illustrate the impact on the Hacat cells with RA, when the cells covered more than 40% of the well, Hacat cells were treated with RA (0.1 μM, 1 μM, 10 μM, 100 μM, National Institutes for Food and Drug Control, Beijing 10704-200318) for 24 h, the expression of DSG1 protein was detected by Western and the cell index was detected using a real-time cell analysis system (RTCA, Roche Diagnostics, Penzberg, Germany) [21]. 

### 2.2. RA Induced DSG1 Deficiency In Vivo

A total of 20 female ICR mice (18 ± 2 g, certificate no.: SCXK 2012-0001), obtained from Beijing Vital River Laboratory Animal Technology Co., Ltd. (Beijing, China), were used in this study. All animals were caged under controlled conditions (temperature: 23 ± 3 °C, humidity: 50 ± 10%, 12 h dark/light cycles). Mice were fed on standard mouse diet and water. The animal care was performed strictly according to the Care and Use of Laboratory Animals of Institutional Animal Care and all experiments were approved by the Animal Ethics Committee of Tianjin University of Traditional Chinese Medicine (Tianjin, China; no. TCM-LAEC2017148). The retinoic acid (RA) (70 mg/kg, Beijing Solarbio Science, 213A031, Beijing, China) suspension was intragastrically administrated for five consecutive days [22]. 

### 2.3. LPS Action on the DSG1-Deficient Hacat Cells

To evaluate whether DSG1 deficiency could make keratinocytes more sensitive, LPS was firstly used as positive stimulation after RA-induced DSG1 deficiency in Hacat cells, the apoptosis rate and the mRNA level of inflammatory factors, including IL-4 and TNF-α, were measured after being cultured with LPS for another 24 h. The Hacat cells in normal group were cultured in the MEM, while the RA group and RA + LPS group Hacat cells were treated with RA (100 μM) for 24 h to downregulate the DSG1 expresion. After overnight incubation, the cells were washed with PBS and incubated with simulants including Lipopolysaccharides (LPS, 20 ng/mL, Beijing Solarbio Science, 102O031) (RA + LPS group) and MEM (RA group) for another 24 h.

### 2.4. SHLI Effect on the DSG1-Deficient Hacat Cells and Mice

To evaluate the effects of SHLI on DSG1-deficient Hacat cells, we added SHLI into the RA pretreatment (100 μM) Hacat cells to make sure whether SHLI could increase the inflammatory factors and apoptosis rate in DSG1-deficient Hacat cells. The normal-group Hacat cells were cultured in the MEM, while the RA group and RA + SHLI group Hacat cells were induced by RA (100 μM). After overnight incubation, the cells were washed with Phosphate buffer solution (PBS) and incubated with simulants, including SHLI (1 μg/mL, 50 μg/mL, 100 μg/mL, Harbin Pharm Group Second Chinese Medicine Factory, 1603609) (RA + SHLI group) and MEM (RA group) for another 24 h.

For animal experiment, the RA group and RA + SHLI group mice were administrated RA suspension (70 mg/kg) for five consecutive days. For comparison purposes, the normal group mice were administrated 0.5% CMC-Na. Then, the SHLI (dissolved in saline, 4 g/kg, eight-fold of clinical equivalent dose) was intraperitoneally injected on the last day, normal mice and RA group mice were administrated a single intraperitoneal injection of saline. The mice skin was removed after injecting SHLI/saline for an hour for HE and Western blot study. 

### 2.5. Cell Viability Analysis

RTCA was used to determinate the impact on the cells with the drugs (RA and SHLI). First, 100 μL of complete medium was added to the 16-wells E-plate for background measurement. Then, the Hacat cell line was seeded in the 16-wells E-plates with a concentrate of 1 × 10^4^ cells/well. The E-plates were placed on the reader in the incubator for continuous recording of impedance to compute the cell index (CI). After 24 h, the cells were treated with RA (0.01 μM, 0.1 μM, 1 μM, 10 μM, 100 μM) or SHLI (0.1 μg/mL, 1 μg/mL, 10 μg/mL, 100 μg/mL, 1000 μg/mL) for another 24 h. The CI was quantified using the RTCA software program version 1.2.1.1002. The data were normalized at the time of starting administrated with drugs. 

The MTT assay was also used in this study. After a 24 h incubation with SHLI (1 μg/mL, 10 μg/mL, 25 μg/mL,50 μg/mL,100 μg/mL, 1000 μg/mL), 10 μL of a 5 mg/mL MTT solution was added to each well and the cells were further incubated at 37 °C for another 4 h. DMSO (150 μL) was added to each well after that, and the plate was placed on a shaker. The absorbance of each well was detected at 490 nm.

### 2.6. Western Blot

Hacat cells or the skin tissue were extracted using a Total Protein and Neuclear-Cytosol Extraction Kit (Beyotime Institute of Biotechnology Inc., Shanghai, China), following the manufacturer’s protocols. Bicinchoninic acid (BCA) assay was used to measure the protein concentration. The proteins were separated by sodium-dodecyl-sulfate polyacrylamide gel electrophoresis, and transferred to a PVDF membrane. Membranes were incubated with antibodies for DSG1 (1:500 dilution, abcam, GR75047-11) and β-actin (1:1000 dilution, Bioss, AE032501) at 4 °C. After overnight incubation, membranes were washed in Tris-buffered saline plus 0.1% Tween-20 for five times. Secondary antibody goat anti-rabbit antibody was diluted to identify the corresponding primary antibodies. Further analysis was carried out by using the Image-pro plus 6.0 (ipp 6.0) to quantify the immunoreactive bands.

### 2.7. Reverse Transcriptase-Polymerase Chain Reaction (PCR)

The mRNA levels of IL-4 and TNF-α were tested by RT-PCR. Reverse transcriptase-polymerase chain reaction was performed as follows. Total RNA was isolated by using the RNA simple Total RNA Kit (Tiangen Biotech Beijing, Beijing, China). And the RNA was reverse-transcribed using FastQuant RT Kit (with gDNase) (Tiangen Biotech Beijing, Beijing, China). Quantitative PCR was performed using SuperReal PreMix Plus (SYBN Green) (Tiangen Biotech Beijing, Beijing, China). Brief complementary DNA amplification conditions were as follows. Cycles of pre-denaturing at 95 °C for 15 min, denaturing at 95 °C for 10 s and annealing/extension at 60 °C for 32 s were repeated 40 times. β-actin/GAPDH was employed for a control reaction. Relative gene expression was calculated using the 2^−ΔΔCt^ method. 

### 2.8. Annexin V-FITC/PI Analysis

An FITC Annexin V Apoptosis Detection Kit (BD Phatmingen, 7303806) was used to detect the cells apoptosis rate. The anchorage-dependent cells, suspended in the cell culture medium, were collected into a centrifuge tube. These cells were resuspended with 1× binding buffer, and AnnexinV-FITC was added into the liquid. The system was incubated at room temperature for 15 min. PI was added subsequently for another 5 min on the ice, protected from light. Flow cytometric was checked out in 30 min. The result was analyzed using FlowJo VX.

### 2.9. Histopathological Analysis

After the mice were intragastric administrated RA (70 mg/kg) for 5 days, SHLI (4 g/kg) was intraperitoneally administrated; one hour later, the skin of back was removed. Some of the skin tissues were trimmed away of the subcutaneous fats and stored at −80 °C for protein quantification. 

The other skin tissues were fixed in 10% neutral buffered formalin and paraffin-embedded, sectioned into 5-micrometer thick, and stained with hematoxylin and eosin (H&E). 

### 2.10. Statistics Analysis

The SPSS software (IBM SPSS Statistics 20.0, SPSS, Chicago, IL, USA) was used for statistical analysis. Quantitative data were presented as mean ± SD., the comparisons were made between multiple groups, statistical significance was determined using ANOVA analysis. *p* < 0.05 was considered statistically significant.

## 3. Results

### 3.1. The DSG1 Protein Expression was Downregulated by RA In Vitro and In Vivo

**Figure 1:**
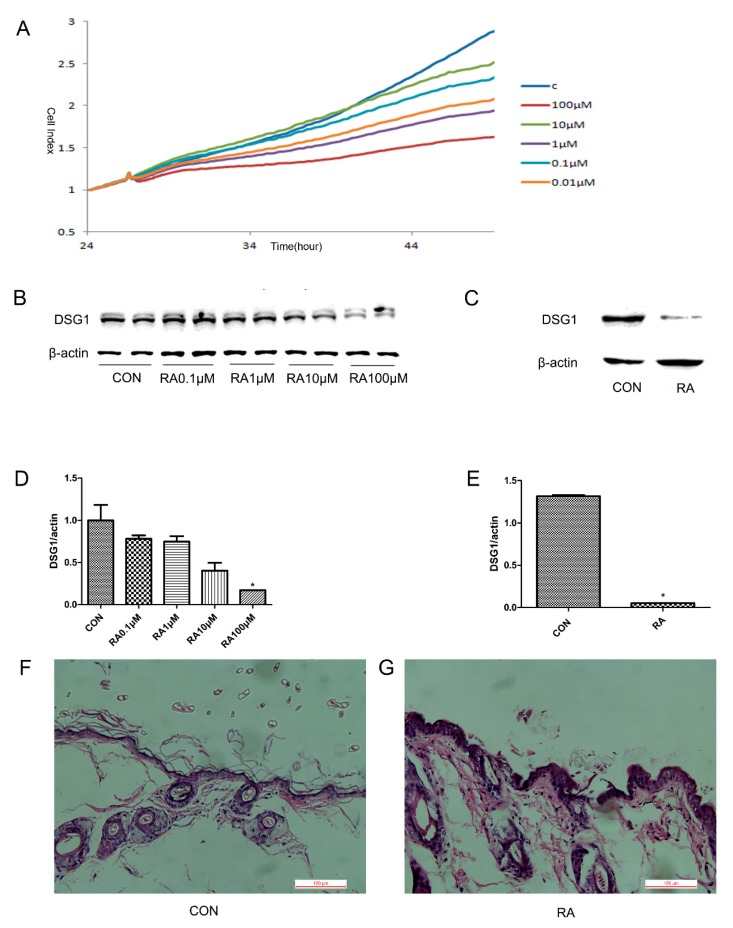
RA downregulated the expression of DSG1 in vitro and in vivo. (**A**) Effects on the Hacat cells index treated with different concentrations of RA (0.01 μM, 0.1 μM, 1 μM, 10 μM, and 100 μM). (**B**,**D**) RA (0.1 μM, 1 μM, 10 μM, and 100 μM) downregulated the DSG1 expression in the Hacat cells. (**C**,**E**) RA (70 mg/kg) downregulated the DSG1 expression in Hacat cells by Western blot. (**F**,**G**) Pathology changes of the skin tissue (HE staining) in mice skin. Data are presented as mean ± S.D, * *p* < 0.05 vs. control.

To understand the effects of RA on DSG1 protein level in Hacat cells and mice skin, RA was used to treat the Hacat cells and mice (Figure 1). According to the RTCA results, the cell viability had no significant change when treated with RA (0.01 μM, 0.1 μM, 1 μM, 10 μM, 100 μM) (Figure 1A, *p* > 0.05). Compared with the normal group, the level of DSG1 expression showed a statistically significant decrease after being treated with RA (100 μM) for 24 h, and presented a dose-dependent manner (Figure 1B,D). 

Furthermore, the DSG1 protein expression of mice skin was also detected after RA treatment (70 mg/kg). The pathological morphology of RA-treated mice skin was first evaluated, and HE results showed that there was no obvious abnormality in the dermis and blood vessels in the skin of RA-treated mice (Figure 1F,G). Western blot results showed that DSG1 expression was downregulated in the skin of RA-treated mice, compared with the normal mice (Figure 1C,E). 

### 3.2. The Deficiency of DSG1 in Hacat Cells Increased the Level of Inflammatory Factors and Apoptosis Rate Induced by LPS

**Figure 2:**
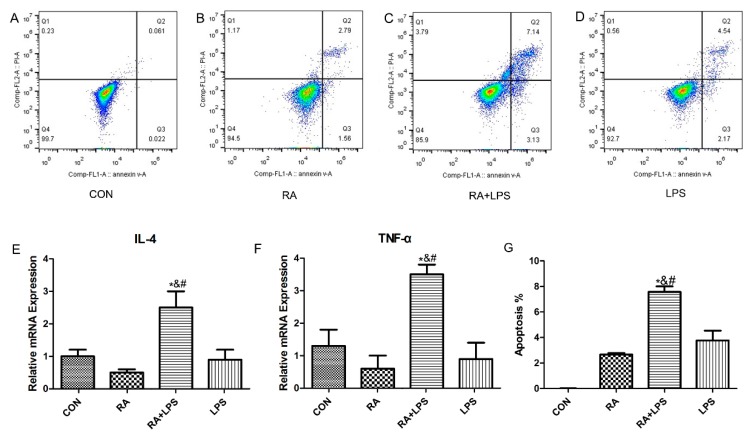
DSG1 deficiency resulted in keratinocytes being vulnerable to injury. (**A**–**D**,**G**) Apoptosis rate analysis by flow cytometry. The mRNA levels of IL-4 (**E**) and TNF-α (**F**) were increased when LPS-reated DSG1-deficient Hacat cells. Data are presented as mean ± S.D. * *p* < 0.05 vs. normal, # *p* < 0.05 vs. RA, and *p* < 0.05 vs. LPS.

LPS was used as positive stimulation to detect the damage-sensitivity of DSG1-deficient Hacat cells. The apoptosis rate and the mRNA level of inflammatory factors, including IL-4 and TNF-α, were measured (Figure 2). RT-PCR results presented limited changes in IL-4 and TNF-α mRNA expression after being treated with single RA or LPS. However, those treated with LPS after RA pretreatment for 24 h showed a significant increase in the IL-4 and TNF-α mRNA levels, compared with the normal group (Figure 2E,F). Besides, treatment with LPS after RA pretreatment for 24 h could induce significant increases in apoptosis rate compared with RA group and LPS group (Figure 2A–D,G). 

### 3.3. The Deficiency of DSG1 in Hacat Cells Increased the Level of Inflammatory Factors and Apoptosis Rate Induced by SHLI

**Figure 3:**
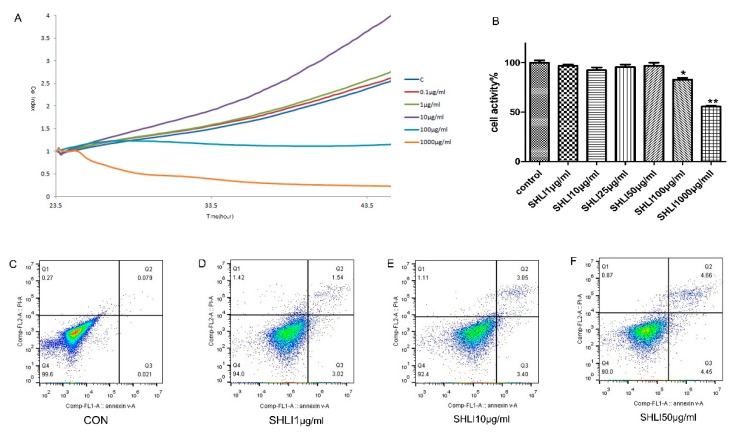
SHLI could not induce apoptosis of the normal Hacat cells. (**A**,**B**) The cell viability analysis of Hacat cell treated with different concentration of SHIL by RTCA (**A**) and Thiazolyl blue (MTT) (**B**). (**C**–**F**) Apoptosis rate analysis by flow cytometry.

**Figure 4:**
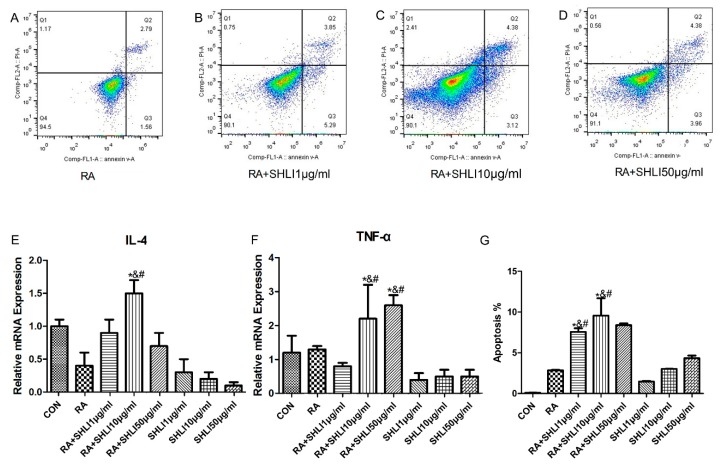
SHLI increased the apoptosis rate and inflammatory factors level of DSG1-deficient Hacat cells. (**A**–**D**,**G**) Apoptosis rate analysis by flow cytometry. The mRNA levels of IL-4 (**E**) and TNF-α (**F**) were increased when different concentrations of SHLI affected the DSG1-deficient Hacat cells. Data are presented as mean ± S.D. * *p* < 0.05 vs. normal, # *p* < 0.05 vs. RA, and *p* < 0.05 vs. SHLI.

To investigate the role of DSG1 in the development of drug eruption caused by SHLI, we first detected the effect of SHLI on the normal Hacat cells (Figure 3). Based on the results of the RTCA (Figure 3A) and MTT (Figure 3B), three different concentrations of SHLI (1 μg/mL, 10 μg/mL, and 50 μg/mL) were chosen for Annexin V-FITC-positive/PI (Propidium Iodide)-positive staining study. Results showed that treatment with SHLI could not lead to a hypersensitive reaction, such as apoptosis (Figure 3C–F) and the release of IL-4 and TNF-α (Figure 4E,F), compared with the normal group. 

To make sure whether SHLI could have the potential to induce drug eruption in DSG1-deficient Hacat cells, the mRNA levels of IL-4, TNF-α and the apoptosis rate were detected after SHLI treatment of the RA pretreated Hacat cells (Figure 4). The results showed that the mRNA levels of IL-4 and TNF-α in the RA (100 μM) pretreatment Hacat cells were significantly increased (*p* < 0.05) after treatment with SHLI, compared with control group, which could not been observed in SHLI or RA group (Figure 4E,F). The flow cytometry results also showed that SHLI could increase the apoptosis rate of the RA pretreated Hacat cells after treatment with SHLI (Figure 4A–D,G). 

### 3.4. SHLI-Induced Cutaneous Adverse Reactions in Mice due to DSG1 Deficiency

**Figure 5:**
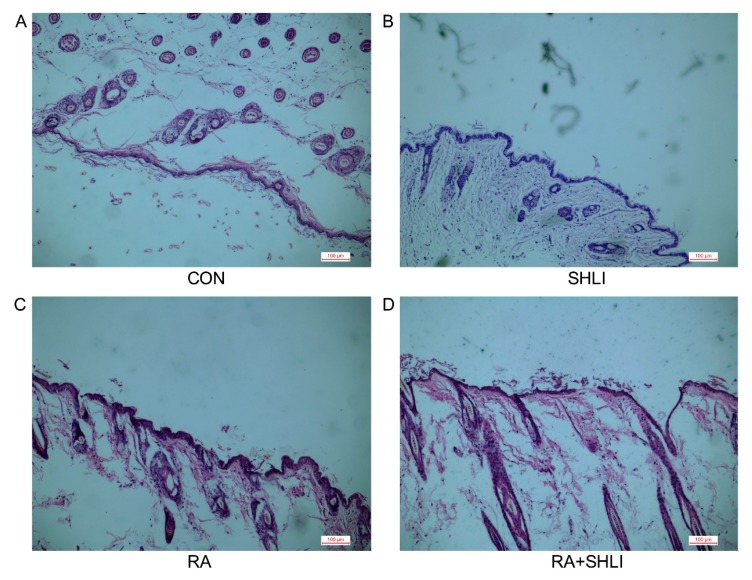
SHLI induced cutaneous adverse reactions because of DSG1 deficiency. (**A**–**D**) Pathology changes of the skin tissue (HE staining) treated with RA and SHLI.

SHLI was intraperitoneally administrated to the mice after RA (70 mg/kg) treated for five days, HE staining was used to detect the pathology changes of the skin tissues (Figure 5). The results of the HE showed no obvious abnormality in the dermis and blood vessels of the normal mice (Figure 5A), and no significant disparity was showed in the single drug group (Figure 5B,C). However, the group that received the combination of RA and SHLI showed thinner stratum corneum of the skin epidermis and a local inflammatory cell infiltration (Figure 5D). 

## 4. Discussion

The present study first identified that the drug eruption of SHLI might be related to DSG1 deficiency, but not the drug itself. Our results showed that the SHLI could not lead to the reactions of drug eruption such as the increase of inflammatory level and cells apoptosis rate in normal Hacat cells. However, after DSG1-deficient Hacat cells were treated with SHLI, extremely different results could be observed: Significant increase in the level of inflammatory and remarkable increase in the cells apoptosis rate. The animal experiment presented a local inflammatory cell infiltration in the skin of those mice with low expression of DSG1 after treatment by SHLI. However, there were no obvious changes when SHLI treatment was applied to the normal mice. Both in vivo and in vitro results suggested that DSG1 played an important role in the drug eruption. These results indicated that DSG1 deficiency was a potential cause of SHLI induced eruptions. 

Drug eruption is one of the most common reactions of the drug reactions. It has been reported that the apoptosis of keratinocytes is the manifestation of drug eruptions [23]. Keratinocytes are an important type of cell in the epidermis. Our study focused on the role of DSG1 during the development and progression of drug eruption. Desmosomes provide a strong adhesion between cells, and the desmoglein is one of the major components of desmosomes. The members of the desmoglein superfamily include four species, desmoglein1, desmoglein2, desmoglein3, and desmoglein4 [24]. DSG1 is expressed in the granular layer, the upper layer of the epidermis, and mucosa squamous epithelium. It is the most important desmoglein, which is responsible for regulating cell adhesion and the differentiation of epithelial cells [8,13]. Kim et al. confirmed that RA could decrease the expression of DSG1 in keratinocytes, based on the observation of mouse skin tissue and a series of experiments [16]. Thus, RA was selected in the present study to downregulate the expression of DSG1. Consistent with previous findings [16], our results showed that RA could downregulate the DSG1 expression in vivo and in vitro. 

It has been reported that DSG1 deficiency and inflammatory mediators can destroy the integrity of the skin epithelium. These reactions can bring about severe dermatitis, multiple allergies, and skin barrier dysfunction [10]. According to Samuelov et al., for human beings, DSG1 deficiency could lead to severe dermatitis, multiple allergies, and metabolic wasting [14]. Therefore, we argued that some of the drug eruption was likely to be caused by DSG1 deficiency. In the present study. to evaluate cell susceptibility, LPS was used as positive stimulation after RA induced DSG1 deficiency in Hacat cells. Our results showed that the apoptosis rate and the mRNA level of inflammatory factors were increased in the DSG1-deficient Hacat cells after treatment with LPS. These results indicated that DSG1-deficient resulted in keratinocytes sensitivity.

The level of inflammatory factors, such as IFN-γ, TNF-α, IL-4, and IL-18 can be elevated in patients with drug eruption [25,26,27,28,29,30]. Therefore, in this study, the expression of the IL-4 and TNF-α, and the rate of apoptosis were chosen to estimate drug eruption. Our results showed that SHLI could increase the mRNA level of IL-4 and TNF-α, and it could also cause the apoptosis of DSG1-deficient Hacat cells. But this phenomenon did not show when treated with a lower or a higher dose of SHLI. We inferred that it might because of the complex chemical composition, some of the ingredients might induce the drug eruption, while other ingredients might have protective effect, further design and study should be carried out to make sure the functions of specific components. Our in vivo results were also showed that mice with low expression of DSG1 were more sensitive after the administration of SHLI. These results indicated that DSG1 mediated the cutaneous eruptions induced by SHLI.

Overall, our study suggested that DSG1 deficiency might be one of the most important reasons of drug induced cutaneous eruption. Since the DSG1 deficiency could be hereditary or induced by pharmacological regulation, the cells and the skin tissues with DSG1 deficiency showed more sensitivity, there would be a high risk of cutaneous reactions when they use SHLI. The present study could also provide a new research strategy on the drug safety evaluation.
